# Development of human pancreatic cancer avatars as a model for dynamic immune landscape profiling and personalized therapy

**DOI:** 10.1126/sciadv.adm9071

**Published:** 2024-07-05

**Authors:** Daniel Hughes, Alice Evans, Simei Go, Michael Eyres, Liuliu Pan, Somnath Mukherjee, Zahir Soonawalla, Frances Willenbrock, Eric O’Neill

**Affiliations:** ^1^ Department of Oncology, University of Oxford, Oxford OX3 7DQ, UK.; ^2^ Medicines Discovery Catapult, Alderley Park SK10 4ZF, UK.; ^3^ NanoString Technologies Inc., Seattle, WA, USA.; ^4^ Department of HPB surgery, Oxford University Hospitals NHS Foundation Trust, Oxford OX3 7DQ, UK.

## Abstract

Pancreatic ductal adenocarcinoma (PDAC) is the most common form of pancreatic cancer, a disease with dismal overall survival. Advances in treatment are hindered by a lack of preclinical models. Here, we show how a personalized organotypic “avatar” created from resected tissue allows spatial and temporal reporting on a complete in situ tumor microenvironment and mirrors clinical responses. Our perfusion culture method extends tumor slice viability, maintaining stable tumor content, metabolism, stromal composition, and immune cell populations for 12 days. Using multiplexed immunofluorescence and spatial transcriptomics, we identify immune neighborhoods and potential for immunotherapy. We used avatars to assess the impact of a preclinically validated metabolic therapy and show recovery of stromal and immune phenotypes and tumor redifferentiation. To determine clinical relevance, we monitored avatar response to gemcitabine treatment and identify a patient avatar-predictable response from clinical follow-up. Thus, avatars provide valuable information for syngeneic testing of therapeutics and a truly personalized therapeutic assessment platform for patients.

## Teaser

Personalized analysis of pancreatic tumor slices provides insight into patient response to therapy.

## INTRODUCTION

Pancreatic ductal adenocarcinoma (PDAC) is the most common form of pancreatic cancer and remains one of the most fatal gastrointestinal tract malignancies. Overall prognosis is poor primarily due to the prevalence of late-stage diagnosis, with 50% of patients presenting with metastatic disease at the time of diagnosis (*1*). Even among those with localized disease at the time of diagnosis, survival rates are still low at 42% (*2*), dropping to 9% overall (*3*). The standard of care for resectable PDAC arising in the head of the pancreas (70% of PDAC cases) involves pancreaticoduodenectomy followed by systemic chemotherapy. The chemotherapeutic regime is dictated by the patient’s overall performance status, whereby patients with a good functional status are commenced on multiagent chemotherapy (FOLFIRINOX), combination gemcitabine and capecitabine, or, if unfit, monotherapy with gemcitabine (*4*). While neoadjuvant therapy is currently used in the context of locally advanced pancreatic cancer, its role in upfront resectable disease has yet to be determined.

Disease occurs through inflammatory signaling leading to acinar cell remodeling and acinar to ductal metaplasia. During this increased plasticity, the cells are increasingly susceptible to mutation and form precursor lesions known as pancreatic intraepithelial neoplasms (*5*). While PDAC is considered a relatively immunologically “cold” tumor type, the immune microenvironment is dynamic during lesion development, moving from an initial pro-inflammatory phenotype to becoming increasingly immunosuppressive (*6*). Along with the shift toward immune suppression, PDAC lesions build up a characteristic desmoplastic stroma through the secretion of collagen by activated cancer-associated fibroblasts (CAFs) (*7*). The fibrous network is formed via tumor signaling through interleukin-6 and tumor growth factor–β, forming a physical barrier thought to hinder the distribution of chemotherapies and prevent immune infiltration (*8*).

Currently, most in vitro therapeutic testing is carried out using patient-derived cell lines, xenografts (PDX), and genetically engineered mouse models (GEMMs). While murine models provide valuable information about the systemic effects of therapeutics, GEMMs fail to capture the genomic heterogeneity of native tumors and PDX models are challenging to establish orthotopically and do not allow study of the interaction of tumor with a functional immune system (*9*). Introduction of patient-derived organoids has allowed in vitro analysis of treatment interactions with three-dimensional tumor structures (*10*) but, similar to xenograft models, does not capture the complexity of the tumor microenvironment (TME) (*11*). Recent efforts move in the direction of incorporating multiple different cell types and vascular mimics to reconstruct tumor complexities (*12*). Patient-derived tumor slices provide a platform through which tumor, stroma, and immune infiltrate can be studied in their native architecture (*13*, *14*). Through this system, therapeutics can be investigated for their impact throughout the tumor, allowing analysis of intrapatient variation in a clinically relevant timeframe.

In this study, we investigate the use of live–patient-derived tumor slices for dissection of the PDAC microenvironment and investigation of therapy response. We have established the use of perfusion culture, which maintains superior cellular fitness and preservation of TME compared to standard static culture. Our analysis of transcriptomic changes induced by a metabolic treatment combination shows the potential of the platform to interrogate treatment responses across cellular compartments, while use of gemcitabine treatment demonstrates the utility of the platform in clinical settings. In clinical application, we refer to the tumor slices as avatars due to their representation of the patient ex vivo. We demonstrate that organotypic tumor slices can maintain viability and provide insights enhancing both therapeutic discovery and precision medicine to improve current standard of care.

## RESULTS

### Perfusion culture maintains a constant metabolic cellular state

**Fig. 1.**
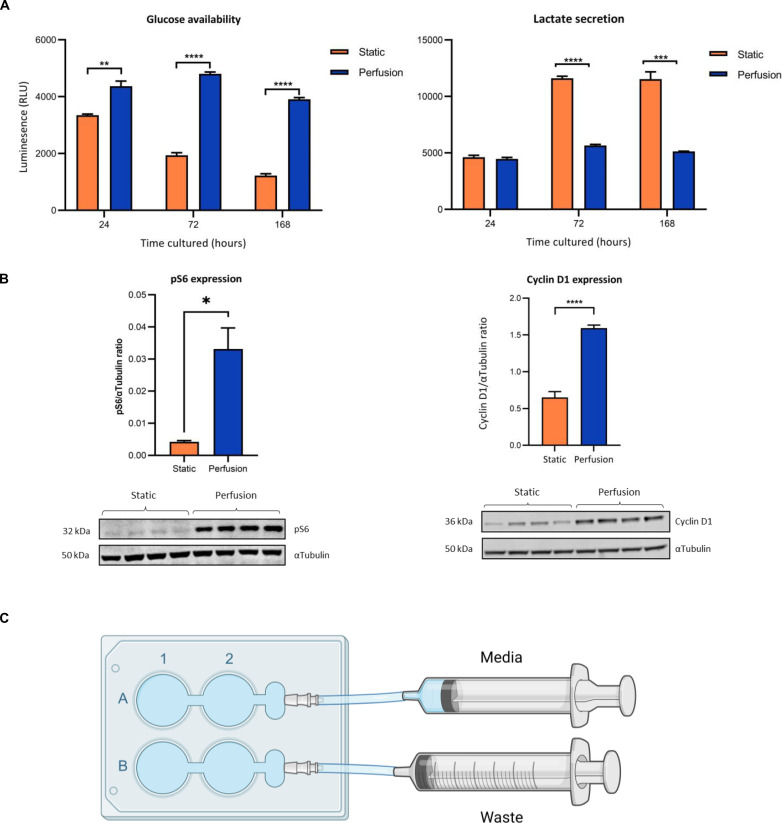
Perfusion culture conditions maintain cell viability. (**A**) Glucose availability (left) and lactate secretion (right) for PSN1 cells maintained under perfusion culture (blue) or static culture (orange) (*n* = 3). (**B**) Immunoblots of p70 pS6 kinase (left) and cyclin D1 (right) for each of the four wells in the culture plate (*n* = 3, error bars ± SEM). (**C**) Schematic of the perfusion dish with equivalent in and out flow rates.

To investigate possible advantages of a system in which cells are continually perfused with medium over the standard static system, we initially cultured the pancreatic cancer cell line, PSN1, under both conditions. For the static culture, a full medium exchange was performed every 72 hours and for perfusion culture, the flow rate was set to ensure that there was a complete medium exchange of the entire perfusion plate by 72 hours (10 μl min^−1^). Media concentration of glucose and lactate were measured at 24-, 72-, and 168-hour time points to determine overall metabolic activity. Glucose concentration in the static culture decreases with time, whereas under perfusion, glucose is maintained at a constant concentration (Fig. 1A, left). Increased glucose uptake indicates enhanced cellular glycolytic activity, and in agreement, we observed reciprocal increase in lactate with standard static culture over time (Fig. 1A, right). Lactate accumulation suggests that following Warburg’s earliest principles for metabolism, static culture shifts PSN1 cells into glucose fermentation, whereas under identical media and oxygen conditions, the constant supply of nutrients and removal of waste products with perfusion allows a more homeostatic culture system. Paradoxically, while lactate can support cell growth and metabolism, lactic acidosis in the microenvironment uncouples mTOR activation and suppresses proliferation particularly in immune cells (*15*) . We find that the excessive accumulation of lactate from PSN1 under 7 days of static culture is associated with an absence of p70 S6 kinase and reduced cyclin D1, whereas these are maintained under perfusion culture (Fig. 1B). This suggests that perfusion represents an improved culture method maintaining cells as metabolically active in terms of proliferation capacity (via Cyclin D1) and protein synthesis (via pS6) and therefore more representative of in vivo physiology than conventional static culture. To ensure responses are independent of plate position relative to input flow, we monitored four separate wells per plate and results are representative (Fig. 1B).

### Perfusion culture preserves the integrity of human tumor slices

**Fig. 2.**
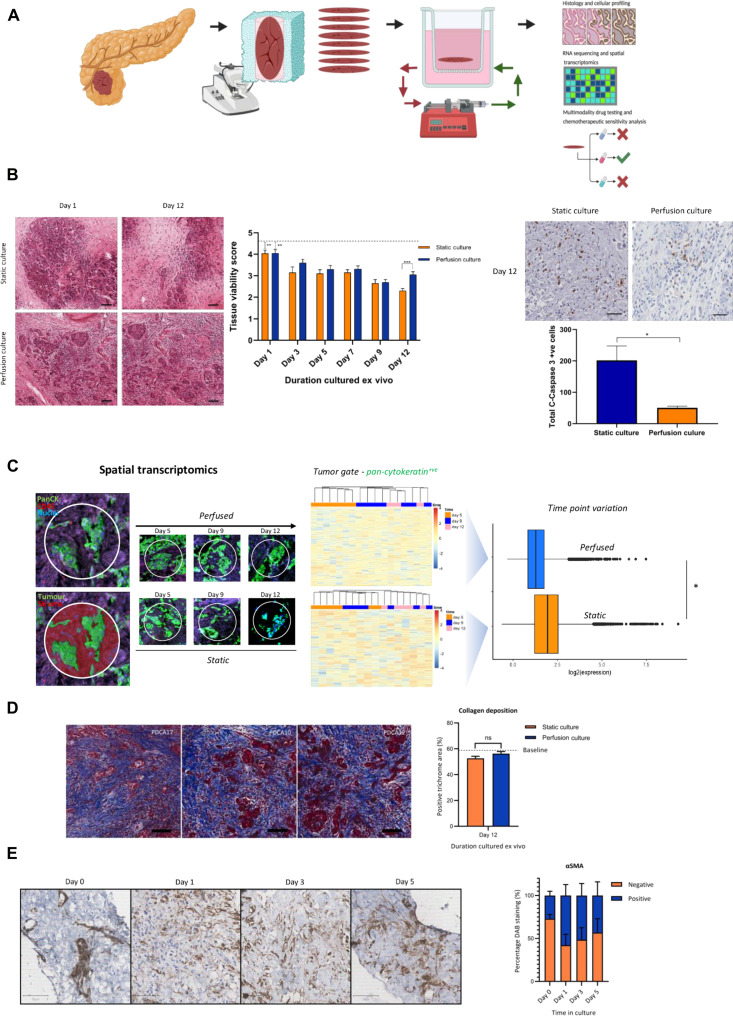
Perfusion culture maintains the integrity of tumor avatars. (**A**) Schematic demonstrating the workflow to establish pancreatic tumor avatar maintenance. (**B**) Histological staining of tumor slices cultured in static and perfusion culture conditions. Images show (left) hematoxylin and eosin staining at days 1 and 12 and quantification over time course and (right) IHC staining of cleaved caspase-3 at day 12, showing elevated cell death under static conditions. (**C**) Spatial transcriptomics of avatar slices. Images show (far left) staining used to gate the samples and (left) improved maintenance of structure over time course in perfusion culture. Heatmaps show the clustering of gene expression data with time (left) and boxplot showing the spread of data (right). (**D**) Representative images of Masson’s trichrome staining for collagen deposition within the extracellular matrix of three independent PDAC avatars at baseline and following 12 days of ex vivo culture. (left) Images taken at 5× magnification (scale bars, 100 μm), (right) quantification of percentage positive area (blue stain) per high power field. Dashed horizontal line represents baseline value (58.6%) (*n* = 3) error bars ± SEM. (**E**) Immunohistochemical images of tumor avatar after culture in perfusion conditions, stained for αSMA (left) and quantification (right) from randomly generated sections (*n* = 3).

We next investigated the effect of perfusion on maintenance of ex vivo tumor slices. We designed a platform for the serial sectioning of resected pancreatic tumor tissue for live-tissue culture, where 250-μm slices were obtained to give a replete cellular microenvironment and within the oxygen diffusion limit to prevent experimental hypoxia (Fig. 2A) (*16*). The platform was engineered to allow comparative multiparameter analysis of the serial sections of a single tumor simultaneously and upon infusion of therapeutics at rates equivalent to delivery to patients. Initially, we assessed the viability of tumor tissue maintained over a 12-day period using both histopathologic scoring and cleaved caspase-3 cell death immunohistochemistry (IHC; Fig. 2B and fig. S2A). Relative to the baseline value (dashed line), a degree of tissue health persists but is maintained to a greater degree when slices are cultured under perfusion conditions. To ensure that baseline values were representative of the tumor, we obtained 250-μm slices from sections separated by >0.5 cm (top, middle, and bottom) and observe similar histopathologic and apoptosis indices (fig. S1, A to C).The TME plays a crucial role in the survival of the cancer cells and is a particular challenge in the treatment of PDAC as it forms the greater part of the tumor mass and is heterogenous, both within a tumor and between patients (*17*). We wanted to assess whether our perfusion culture conditions were sufficient to also maintain the integrity of the other main components of the TME. We used spatial transcriptomic analysis to interrogate the gene expression of tumor and stromal compartments maintained under perfusion. A heatmap of tumor pan-cytokeratin^+ve^ gated expression data demonstrates that overall transcription is more stable under perfusion, whereas tissue slices in static culture degenerate over time with increased variability (Fig. 2C). This was also supported by the pan-cytokeratin^−ve^ stromal gate where similar transcriptional stability was maintained for perfused conditions (fig. S2B). This strongly suggests that tissue slices maintained under perfusion retain their integrity and are transcriptionally stable over time. The stromal compartment was directly analyzed for collagen deposition and CAFs using α–small muscle actin (αSMA) to detect fibroblast activation. Both were stable over time, without excessive outgrowth of CAFs or increased extracellular matrix deposition (Fig. 2, D and E).

### Tumor slice culture gives valuable insight into immune infiltrate interactions

**Fig. 3.**
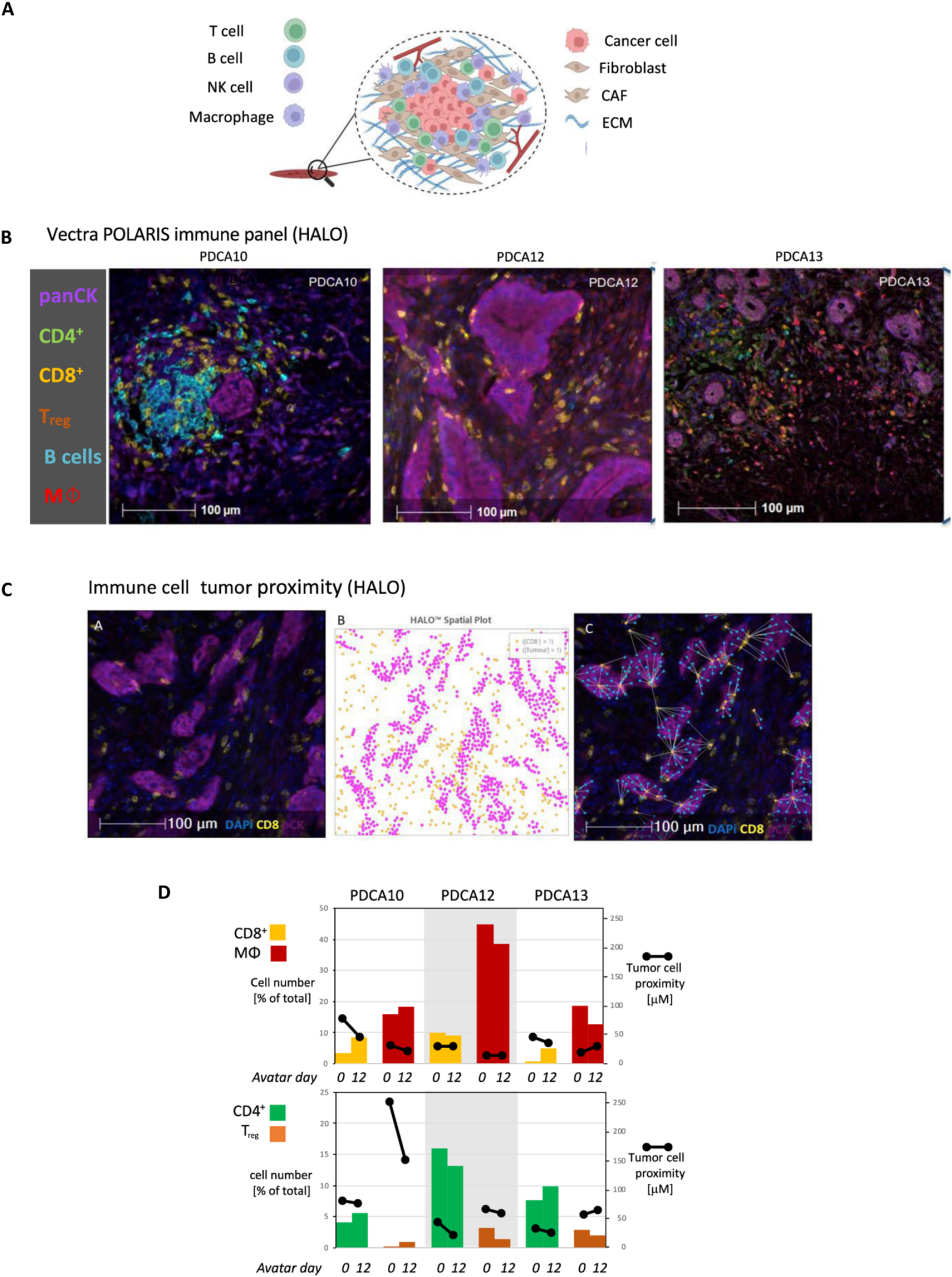
Immunofluorescent staining of the immune population in PDAC avatars. (**A**) Schematic demonstrating the heterogeneity and cell populations present in a pancreatic tumor. (**B**) Images of immunofluorescent staining of avatars from three different patients (PDCA10, PDCA12, and PDCA13) demonstrating the panel used to detect immune cells. (**C**) Example use of HALO software to analyze immune cell proximity to tumor cells. (**D**) Summary of immune infiltrate cell numbers and proximity measurements for PDCA10, 12, and 13.

Increasing evidence has shown that the immunological status of tumors can affect directly upon patient outcomes (*18*). We record variation in the composition of the immune infiltrate between patients, which cannot be accounted for in most preclinical models. We used patient tumor slices to explore individual patient immune landscapes using multiplex immunofluorescence (IF) using a panel that detects CD4^+^ T cells, CD8^+^ T cells, regulatory T cells (T_regs_), macrophages, and B cells (Fig. 3A and table S1). Analysis of the immune landscape of tumor slices of three patients (PDCA10, PDCA12, and PDCA13) over 12 days was used to allow sufficient time for expansion or modulation of immune phenotypes and any micromigration toward tumor cells within the tissue slice. This allows total measurements of intrapatient heterogeneity (Fig. 3B, fig. S3A, table S2) and cell proximity measurements of regulatory and effector immune cells and between effector immune and tumor cells (Fig. 3C and fig. S3B). As immune populations in the perfused slices are stable throughout the 12-day time course (Fig. 3D and fig. S3A), we reasoned that the functional state of the local immune environment could be inferred from these measurements of proximity indicating cellular interactions (fig. S3B). This suggests that our perfused system maintains the tumor slices in a state that recapitulates that of the original tumor and therefore of potential utility for interrogating tumor responses ex vivo.

### Patient avatars have varied immune neighborhoods

**Fig. 4.**
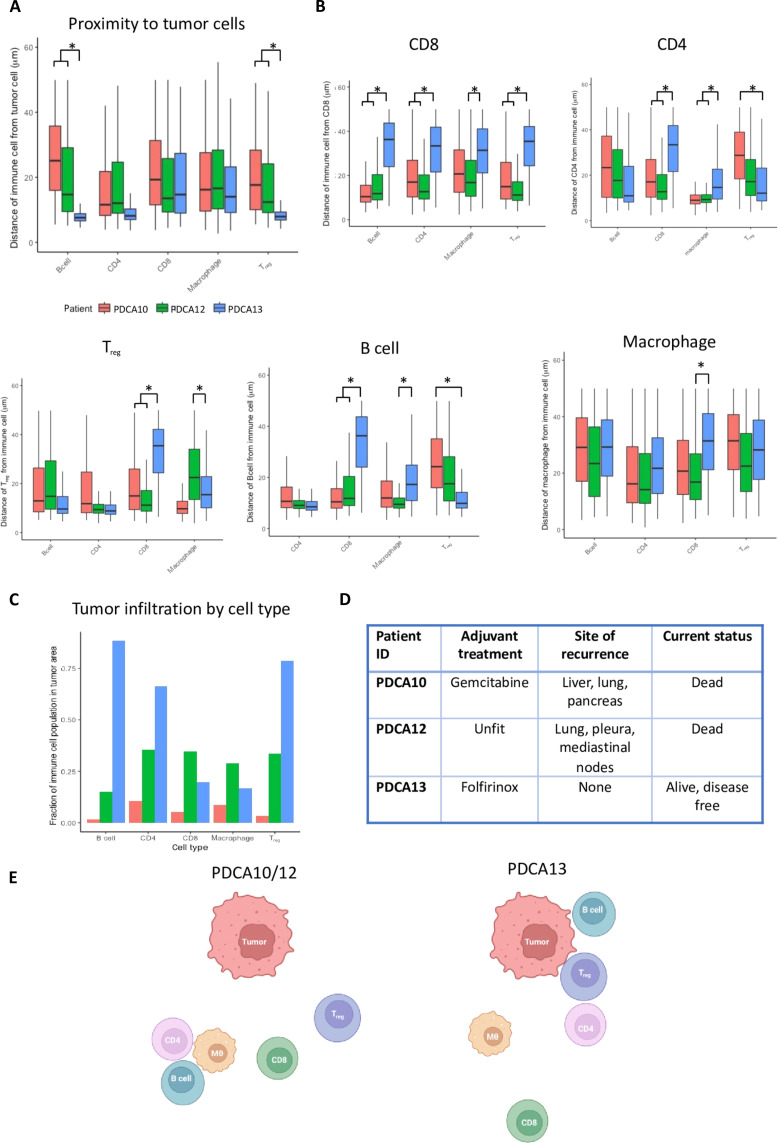
Spatial analysis of the immune population in PDAC avatars. (**A**) Proximity of immune cells to tumor cells in patients PDCA10, 12, and 13. Nearest-neighbor analysis was performed on cells that were within 50 µm of tumor cells. Box and whisker plots display the median, quartiles, and range of the data. Significance is inferred from a Cohen *d* statistic for the Welch test of >0.8. (**B**) Proximity of immune cells to other immune cells. Analysis as for (A). (**C**) Immune cells residing within delineated tumor cell areas as a fraction of the total population of that cell type within the avatar. Cell numbers are given in table S2. (**D**) Clinical outcome for patients PDCA10, 12, and 13. (**E**) Schematic showing the relationship between immune filtrate and tumor cells for each of the patients, as interpreted from the nearest neighbor analyses.

We next assessed the utility of determining immune neighborhoods between patients’ avatars by exploring proximity between immune cells at baseline, similar to tumor cells above. Using “proximity” calculations in the HALO software, we determined cell-cell distances <50 μm and used “size effect” >0.8 as a measure of significance, using Cohen’s *d* statistic for the Welch test that adjusts for different cell population sizes and variances (*19*). We used baseline measurements of patient avatars, identifying some commonalities such as CD4^+^ T cell proximity to tumor cells and to B cells (Fig. 4A). Despite common features of patient immune neighborhoods, the avatars also reveal that B cells and T_regs_ are closer to tumor cells in PDCA13 compared to PDCAs 10/12 (Fig. 4A). In addition, CD8^+^ T cells in PDCA13 were more distant from all other immune cells than those in PDCA12 and PDCA10 (Fig. 4B). We observed that there were no significant differences between the immune neighborhoods of PDCA10 and PDCA12, while PDCA13 displayed a number of variations. We see clustering of B cells, T_regs_, and CD4^+^ T cells near the tumor in PDCA13 with distinct CD8^+^ T cell exclusion, while PDCA12 shows a B cell, macrophage, and CD4 clustering, which is less tumor proximal (Fig. 4B). We again analyzed the effects of perfusion in this context, noting that there was no change in the proximity of immune cells relative to tumor or to each other between days 0 and 12 (fig. S3). To support this analysis, we examined IF images to investigate the numbers of cells residing within the tumor cell areas (Fig. 4C and table S3). In this analysis, the peritumoral area was defined as up to 15 μm outside the interface, while the intratumoral area was the entire area of a cluster of tumor cells. Across the three patient avatars at baseline, we observed variation with the intratumoral immune cell infiltrate. The PDCA13 avatar had the greatest proportion of intratumoral B cells, T_regs_, and, to a lesser extent, CD4^+^ T cells in comparison to PDCA10 and PDCA12, which agrees with our proximity measurements. We note that patient survival also differed along the same lines as observed with the IF results, with patient PDCA13 surviving for considerably longer than the other two patients (Fig. 4D). A schematic representation of each patient tumor indicates how the results can be interpreted to infer individualized immune neighborhoods (Fig. 4E) and can be valuable spatial information expanding on neighborhood inferred from single-cell RNA sequencing data (*20*). Thus, such analyses of ex vivo tumor slices as representative patient avatars may give valuable insight into the role of the immune infiltrate in patient survival.

### Treatment of patient tumor slices with metformin and ascorbic acid modifies stromal and immune cells

**Fig. 5.**
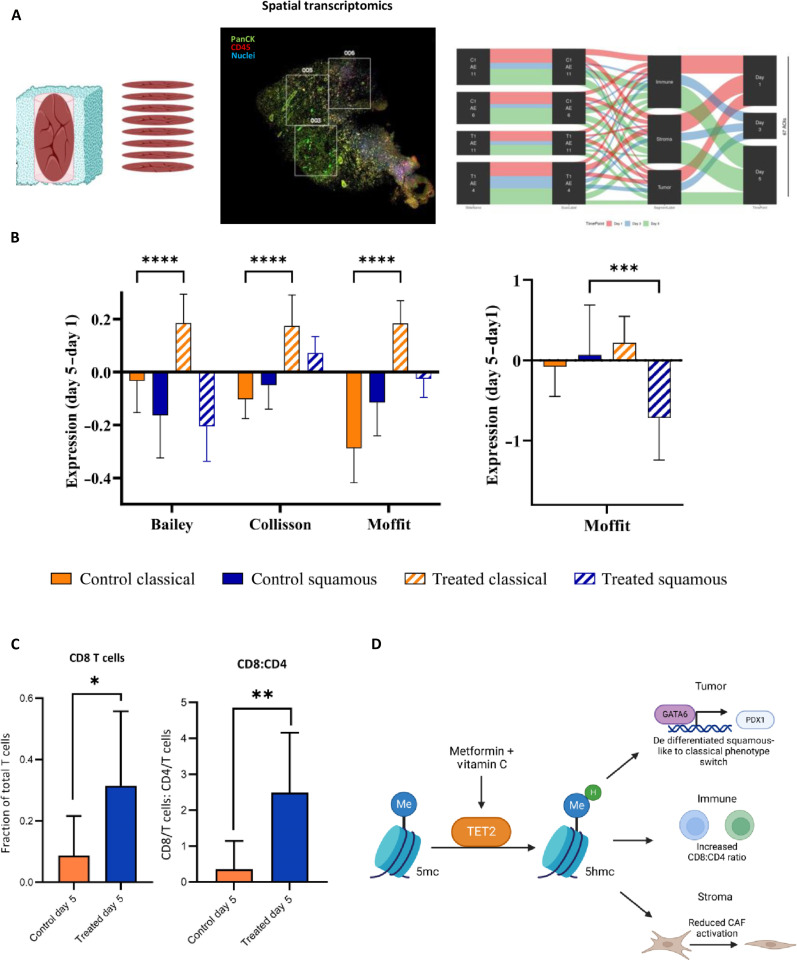
Effect of 5 day treatment with ascorbic acid and metformin on avatar components. Avatars were perfused for 5 days with 20 μM metformin and 100 μM ascorbic acid as described in Materials and Methods. (**A**) Schematic depicting the spatial transcriptomic analysis of avatars. For each condition, two sections were analyzed using >6 regions. (**B**) GSVA analysis on tumor component using three published gene sets, which define the classical and squamous-like phenotypes, with two-way ANOVA multiple comparisons for the tumor types and one-way ANOVA for the stroma. (**C**) GSVA analysis on stromal component of avatar using the published gene set for the classical and activated phenotypes. (**D**) Results from CIBERSORTx deconvolution of immune component to show effect on CD8 T cells. (**E**) Schematic depicting the effect of treatment on each avatar component.

We have previously demonstrated in a preclinical study that treatment of tumors with a combination of metformin and ascorbic acid induces phenotypic change of pancreatic tumor cells from aggressive squamous-like to classical, therefore rendering cells potentially more susceptible to chemotherapy (*21*). It has been reported that the stromal component of PDAC tumors can also exist in an activated form, which is found in, and assists in maintenance of, more aggressive tumors (*22*). We therefore investigated the effect of metformin and ascorbic acid on the TME by perfusing a tumor slice with the drug combination and analyzing the effect on cell phenotype using spatial transcriptomics. Gene expression data for both the tumor and stroma sections were analyzed using gene set variation analysis (GSVA) with the published gene sets that describe the squamous-like and classical phenotypes (*22*, *23*, *24*). The means of the GSVA scores for each condition and for each gene set were then further processed by subtracting the day-1 values from those of day 5 and results plotted for control and treated samples (Fig. 5, A and B). For each of the three gene sets used to analyze tumor cells, there is a marked increase over time in the representation of the less-aggressive classical gene set in the treated samples compared to the control samples and that there is a greater representation of classical over squamous-like genes. This confirms our previous observations on the effect of metformin and ascorbic acid. When we analyzed the stromal compartment using the gene set for the stromal subtypes (*22*), we observed an increase in the form of stroma associated with less aggressive tumors after the 5-day treatment regime and a decrease in the more aggressive activated stroma (Fig. 5C). Thus, treatment alters the phenotype of both tumor cells and associated stroma.

Last, we investigated the effect of the drugs on the immune infiltrate of our avatars. To do this, we deconvoluted the gene expression data for the immune compartment using the CIBERSORTx software and followed change in numbers of different types of immune cells at day 1 compared with the later time points of days 3 and 5. We observed an increase in the numbers of CD8 T cells after treatment with metformin and ascorbic acid (Fig. 5D), an effect that was not seen for any other immune cell population (fig. S4). The elevated ratio of CD8:CD4 T cells was also observed through IF staining of the sections (fig. S5), which could be indicative of a loss of CD4 or CD8 cell proliferation and requires a deeper exploration of lymphocyte phenotypes. In any case, these data strongly suggest that the metformin and ascorbic acid combination modifies not only the tumor but also the key components of the TME to enhance the therapeutic effect of the combination (Fig. 5D). Use of tumor avatars to dissect the effect of potential drugs on all aspects of tumor biology is a powerful addition to currently available techniques.

### Drug testing on avatars can allow rapid, efficient personalized treatment response analysis

**Fig. 6.**
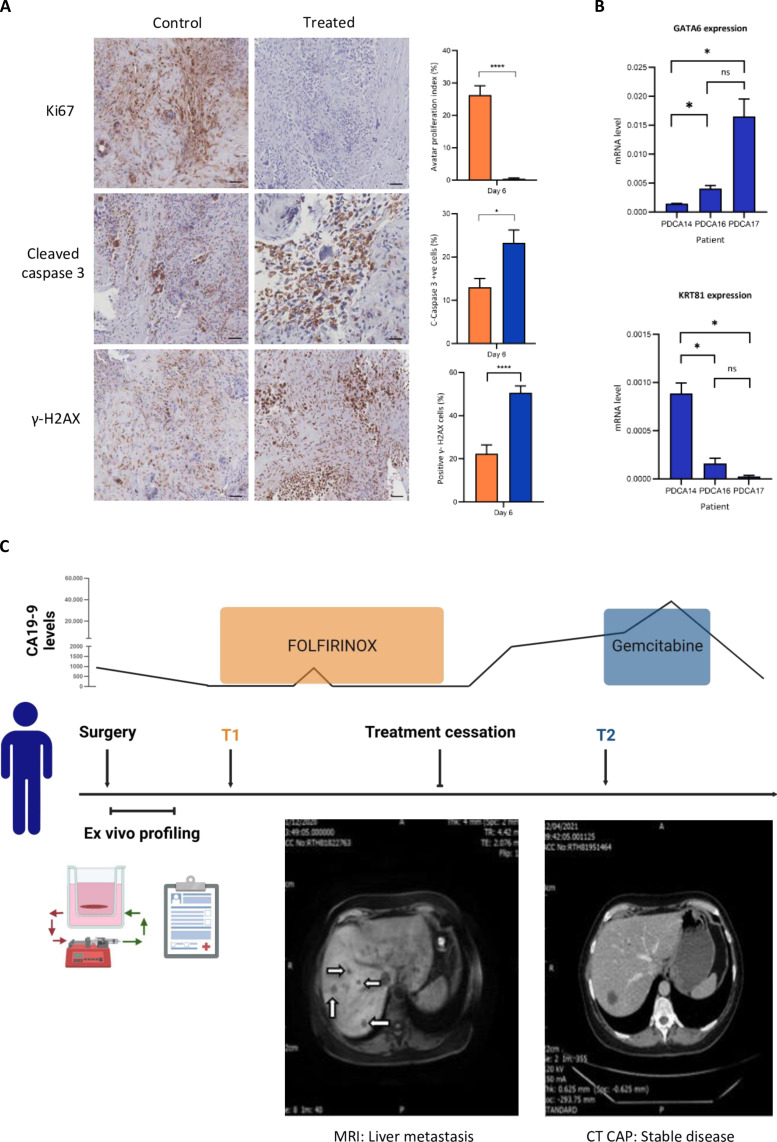
Use of patient avatars in precision therapy. (**A**) (left) Immunohistochemical staining of PDCA14 avatar 6 days after gemcitabine treatment (perfused for 1 hour at a concentration of 250 μM): top, Ki67; center, cleaved caspase-3; bottom, γ-H2AX activation. Images taken at 10× magnification; scale bars, 50 μm. Quantification (right) using randomly generated sections, *n* = 3 slides. Data shown are mean ± SEM. (**B**) Quantification of *GATA6* and *KRT81* gene expression in baseline samples of tissue from patients PDCA14, PDCA16, and PDCA17 using qPCR. (**C**) Schematic of PDCA14’s clinical journey from diagnosis showing tumor marker, CA19-9, trends over time presented as a line graph and times at which treatment was given along with cross-sectional imaging of pre- and post-gemcitabine treatment.

A key aim of the current work was to use avatars as a precision medicine tool to ascertain which adjuvant treatment will most benefit each patient. The procedure is ideal for this since chemotherapy starts approximately 3 months after surgical resection. Thus, avatars can be tested with potential drugs and results analyzed in time to select further treatment. To assess whether our system can be used in this way, we tested avatars from three patients by perfusing them with gemcitabine for 24 hours and culturing for a further 5 days, followed by fixation and IHC to investigate levels of proliferation and cell death (Fig. 6A). We also isolated RNA from baseline samples of each section and assessed *GATA6* and *KRT81* gene expression by quantitative polymerase chain reaction (qPCR; Fig. 6B). The low expression of *GATA6* and high expression of the *KRT81* genes in patient PDCA14 suggest that this tumor is squamous-like and therefore, a more aggressive subtype. PDCA16 and PDCA17 show the opposite expression profile and therefore are likely to be classical tumors. Squamous-like tumors are reported to have a greater sensitivity to gemcitabine therapy, which we observed within our drug-treated avatar cohort; PDCA14 showed decreased cell proliferation and increased cell death (Fig. 6A), whereas PDCA16 and PDCA17 showed no response to treatment (fig. S6). The clinical outcome of the patients (taken at 18 months) correlated with our avatar observations. PDCA14 was treated with surgery, adjuvant FOLFIRINOX, and palliative gemcitabine; PDCA16 with neo-adjuvant FOLFIRINOX, surgery, and no further chemotherapy; and PDCA17 with surgery and no further chemotherapy. For PDCA14, the FOLFIRINOX regime may have initially secured systemic control over the disease. However, over time, the therapeutic effect was lost, and tumor outgrowth and metastases occurred. A comparison of tumor behavior after the administration of palliative gemcitabine shows that there was not only a notable reduction in the Ca19-9 tumor marker but also 8 months of stable metastatic disease with no radiological evidence of progression (Fig. 6C). This is very different from the tumor behavior before gemcitabine therapy, where the disease burden had increased. The lack of increase in the metastatic burden since completing palliative therapy implies sensitivity of the tumor to gemcitabine in agreement with our observations with the patient avatar. Information concerning the stage, lymph node involvement, and survival of the patients was also collected (table S5). Patient PDCA16 responded well to neoadjuvant FOLFIRINOX treatment and did not receive any further chemotherapy during the follow-up period. Patient PDCA17 also did not receive adjuvant therapy. In both cases, on the basis of our results, gemcitabine would not have been expected to be effective (fig. S6). The correlation observed between avatar behavior and patient response makes this a viable option for assessing which treatments would be suitable for each patient and is therefore a rapid, economical method for personalizing PDAC treatment.

## DISCUSSION

There is increasing evidence that the entire TME contributes to progression therapeutic responses of cancer (*24*). This is particularly important in PDAC, which has not only a heterogeneous microenvironment but in most cases forms the major component of the primary tumor. Thus, the role and interactions of each component of the tumor needs to simultaneously be addressed in the assessment of candidate drug efficacy. In addition, tumor properties vary considerably between individuals, most notably in the existence of different transcriptional subtypes of tumor and stromal cells with differing treatment responses.

While organotypic slices provide previously unidentified insights, it is important to consider the limitations imposed by structural variation throughout the tumor. There is a degree of heterogeneity between slices necessitating careful selection of response readouts. Ideally, replicates of three slices would be used across experimental conditions to account for such heterogeneity. Although this is a limitation in terms of potential experimental conditions, intratumoral heterogeneity must be accounted for when assessing therapeutic efficacy. This aspect of tumor slices is unique to the system and cannot be recapitulated in organoid or murine models.

Tumor slices allow an insight into the different tumors of a diverse patient population. Because of the invasive nature of sample collection (in particular, in cancers such as PDAC where rapid progression means that biopsy is not possible before surgery) these tumors represent a single time point in tumor progression. It is therefore important to investigate responses seen in slices in additional models such as GEMMs to obtain a longitudinal view of cancer progression. This is also important when considering the systemic effects of treatment, which requires use of in vivo models.

In this work, we aimed to establish a system for studying PDAC that preserves the tumor cells within their native microenvironment for sufficient periods to reliably assess the efficacy of treatment regimes. A number of studies have confirmed that maintenance of patient tumor sections could provide information about many aspects of tumor biology (*13*, *14*, *25*–*28*). Most of the published work has consisted of simply maintaining tumor cultures on scaffolds under static conditions, although a recently reported method uses a perfusion air culture of ovarian cancer slices in which slices are exposed to air on the upper surface and medium perfused over the lower surface (*29*). The development of our avatar-perfusion system stemmed from our initial observations that cells grown in static culture are metabolically unstable, important for tumor and stromal cells but vital when considering viability of immune cell populations that are more sensitive to stress. In tumor slices, all three major cellular components, i.e., tumor, stromal, and immune cells, remained viable and stable over a 12-day time course, compared to static conditions with identical medium exchange over 72 hours. The 12-day window was selected to span the average length of hospital stay following resection (7 to 12 days), enabling the eventual coordination of appropriate treatment pathways in line with patient postsurgical recovery. By using a multiplexed IF using a panel, we were able to distinguish broad immune populations cells (B cells, CD4^+^, CD8^+^, macrophages, and T_regs_), which allowed an unparalleled simultaneous analysis of populations and relative proximity within a human tumor ex vivo.

Given that the patients varied in tumor staging and therapeutic regimen before avatar profiling, a larger study would need to be conducted to fully interrogate the impact of immune neighborhoods on overall survival. However, a major advantage for this method is the ability to identify discrete variations between individual patient immune environments, e.g., we see more peritumoral localization of B cells and T_reg_ in patients PDCA10/12 compared to the longer-surviving patient PDCA13, where B cells display closer proximity to tumor cells. An advantage for this level of information is that this, together with the unexpectedly lower levels of CD8^+^ T cells in PDCA13, could support the role of B cells in better responses and overall survival. Emerging evidence suggests that infiltrating B cells serve as a prognostic marker for long-term survival for patients with PDAC (*30*, *31*) and may facilitate cross-presentation of antigens to proximal CD4 T cells leading to up-regulation of T helper cell 1 (T_H_1) and T_H_2 responses (*32*). Our approach is also able to discern discrete variations in T_reg_–CD8 T proximity between the avatars, showing increased distance between, which could be interpreted as a lack of CD8 suppression by T_reg_ signaling (*33*). Macrophages were the most abundant immune cell infiltrate within all three patient avatars in agreement with published data (*34*), and we see greater separation from CD8 T cells in PCDA13. Additional monitoring of immune subpopulations, e.g., M1 versus M2, is readily achievable with expansion or redesign of immune markers in the IF panel.

We view the main strength of this approach as the dynamic monitoring of immune diversity within the PDAC avatars. The advantage is a unique ability to test immunotherapies in a preclinical human setting and provide proven personalized treatment strategies in real time for patients. For example, in the context of a patient with a high intratumoral immune infiltrate, therapies should be tailored toward overcoming local checkpoint immune blockage and preventing immune cell exhaustion, whereas in cases with low intratumoral immune environment, a distinct strategy may require myeloid suppression to boost infiltration of cytotoxic lymphocytes.

We tested the system to study the potential of a previously tested combination of ascorbic acid and metformin that promotes a more differentiated tumor phenotype (*21*), to augment the TME. By targeting metabolism, this combination actively restores cell phenotype by restoring global hydroxymethylation and reverting the more aggressive, dedifferentiated basal-like form of PDAC to better differentiated *GATA6* expressing classical phenotype (*21*, *35*–*38*). As the perfusion system stabilizes metabolism, we could test the avatars for the ability to assess the effect of this therapy on tumor cells and other cell populations in the TME simultaneously in situ. As expected, the metabolic therapy promoted redifferentiation of PDAC phenotype but additionally converted stroma subtypes from activated cancer associated to more normal phenotype found in less aggressive tumors and supported an elevated proportion of CD8^+^ T cells. This expansion was not coupled with an increase in tumor cell killing, indicating an insufficiency in activity of these cells despite their elevated proportions. Whether this later immunological effect was due to de novo expansion in the avatar requires further analysis but demonstrates the robustness of the platform for investigation of a replete TME.

A key aspect of our system is that therapeutics can gradually be perfused over the avatars, providing a more physiological application process than the single dose provided in static culture systems. To assess the ability of the system to predict patient response to chemotherapy regimens, we tested avatar response to gemcitabine treatment for three patients and subsequently correlated this to patient outcome and PDAC subtype. Avatar PDCA14 showed a specific increase in cell death with gemcitabine, which accurately predicted palliative gemcitabine response for patient PDCA14. Notably, while the avatar was derived from the primary tumor, liver metastases that developed after 11 months appeared to retain the phenotypic sensitivity of the primary tumor. Thus, it appears that as there is little phenotypic distinction between primary and disseminated tumors (*39*), those avatars could determine second-line therapy for patients with metastatic disease. Another practical application is in aiding decisions for alteration of on-treatment systemic therapy. For example, patient PDCA16’s tumor was initially locally advanced but was successfully downstaged following a good response to FOLFIRINOX. If the patient’s disease were to recur, a treatment plan would require a decision to restart the neoadjuvant regime or commence a different therapy for palliation. On the basis of the data generated from the avatar, no therapeutic response was noted with gemcitabine. As such, an argument that could be made is that palliative gemcitabine should not be commenced, and that the neoadjuvant regime (FOLFIRINOX) should be recommenced considering the previous response.

In future work, we will build upon our existing knowledge of the longevity of the tumor slices to analyze the matrix stability. An important aspect of future work will be incorporating hypoxia into the system as most tissue exists within an environment of 3 to 9% oxygen (*40*) and pancreatic tumors are often particularly poorly vascularized (*41*). Replicating these lower oxygen levels to observe the effect on cell viability, tumor cell phenotype, and response to therapy will be integral in forming an accurate representation of tumors ex vivo.

We aim to characterize the ability of tumor slices to predict individual response to investigational therapies. We will leverage the presence of resident immune cells to provide insights into microenvironmental factors influencing immunotherapy efficacy. We will additionally explore metabolomic signatures due to mounting evidence that metabolic factors play a major role in cancer progression and treatment responses (*42*–*44*).

Thus, ex vivo treatment of patient avatars shows promise as a potential model to deliver precision therapy. Using a diverse range of downstream analyses allows investigation of individual patient immune cell neighborhoods, cancer subtype, and treatment susceptibility. The data illustrate that patients who share a common cancer diagnosis actually have distinct and individual tumor behaviors and as such, a generalized treatment approach is inappropriate, and regimens should be specific for each individual patient. For any clinical benefit to be realized, real-time assessments of therapeutic effects must be made within a viability window, prolonged by a perfusion culture system. Thus, PDAC avatars provide a versatile platform to allow such treatment personalization, along with evaluation of investigational therapies.

## MATERIALS AND METHODS

### Acquisition of tissue and blood samples

The study (REC number 19/A056) was approved for the collection of tumor and healthy pancreatic tissue by the Oxford Radcliffe BioBank. Collection of the specimens was supported by the Oxford Centre for Histopathology Research. All patients recruited to the study provided written consent confirming voluntary participation and permission for tissue donation for research. Biopsy punch samples (5 mm diameter) were obtained by the pathologist at the John Radcliffe hospital following surgery provided that clear surgical margins could be determined.

### Sectioning and culture of live-tumor slices

Samples were transported on ice in unsupplemented low-glucose Dulbecco’s minimum essential medium (LG DMEM) media before suspension in agarose scaffolds. Following a manual wash in LG DMEM media, biopsy punches were suspended in 4% low-geling-temperature agarose and cooled. The agarose scaffold structure was generated by melting the solution and suspending the slice in a small embedding mold. Sections (250 μm) were generated using the Leica VT1200 vibratome (blade angle, +21°C; speed, 1.5 mms^−1^; and amplitude, 2 mm) in a bath of ice-cold phosphate-buffered saline (PBS) and transported in LG DMEM media on ice.

Alvetex perfusion plates (REPROCELL) were used to conduct dynamic perfusion experiments, with syringe pumps maintaining a constant flow of 10 μl min^−1^. The apparatus was assembled within a sterile tissue culture hood. To construct the circuit, 60-ml syringes were connected to silicone tubing and flushed with 70% ethanol before washing in unsupplemented LG DMEM media. Alvetex 12-well tissue culture inserts were activated in 70% ethanol for 2 min before washing in LG DMEM media. Long-term culture of tissue slices was conducted using LG DMEM complete pancreatic medium (see reagents table) at +37°C and 5% CO_2_.

### Treatment of slices in perfusion culture

All ex vivo treatment of avatars was commenced on day 0 (day of tumor acquisition). Systemic chemotherapy was prepared in dimethyl sulfoxide. Drug delivery commenced after the avatars had been created and subsequently placed in the perfusion plate, via an inflow circuit comprising a syringe and tubing, attached to the perfusion plate and connected to the perfusion pump. After the intended time period for drug delivery, the infusion was stopped, and the perfusion plate removed from the incubator and placed within a tissue culture hood. The inflow circuit was removed and the inflow channel on the perfusion plate temporarily occluded using a 2-ml syringe. An inflow circuit was then created and the syringe filled with culturing media. The inflow circuit was primed with media (from the attached syringe) to remove any air bubbles present in the tubing before attachment to the perfusion plate to ensure that there was a constant column of media from the syringe and the tubing to the perfusion plate. For treatment with metformin and ascorbic acid, 20 μM metformin and 100 μM ascorbic acid were perfused for 5 days, with freshly made-up solutions applied each day. Gemcitabine concentration was perfused for 1 hour at a concentration of 250 μM.

### Cell culture

Pancreatic cell lines were cultured in DMEM media supplemented with 10% fetal bovine serum 1% l-glutamine and 1% penicillin-streptomycin (100 U ml^−1^). Cells were split at 90% confluency using 0.25% trypsin-EDTA solution (or TrypLE solution if examining surface epitopes). Cells were frozen in freezing media containing 90% (v/v) fetal calf serum and 10% (v/v) dimethyl sulfoxide and stored in liquid nitrogen.

### Metabolic substrate profiling

Glucose and lactate analysis was performed using the Promega Glucose-Glo assay and Lactate-Glo assay kits according to the manufacturer’s protocol. Cells were seeded in six-well tissue culture plates and four-well perfusion plates at equivalent densities with starting number optimized to reach confluency at the end of the sampling period. Samples of media were taken every 48 hours before manual media change of the static plate. Media samples were diluted as per the manufacturer’s instructions and stored at −20°C. The reaction was carried out as per the protocol and luminescence was read using the POLARstar Omega Microplate Readers.

### Western blot

Cell lysates were obtained from adherent cells after washing with cold PBS by addition of 200 μl of RIPA lysis buffer with cOmplete protease inhibitor (Roche) and PhosSTOP phosphatase inhibitor (Roche) and 5-min incubation on ice. A cell scraper was used to lysates, which were then centrifuged at 14,000*g* for 15 min, supernatants were transferred to fresh Eppendorf tubes for analysis. Protein was quantified using the Pierce BCA Protein Assay Kit.

Gel electrophoresis was performed using 10% bis-tris gels with 50 μg of protein in each well. Samples were diluted in RIPA buffer with NuPAGE LDS sample buffer and 1 mM dithiothreitol before running at 150 V for 90 min in MOPS buffer. Following electrophoresis, transfer was achieved using a polyvinylidene difluoride membrane Immobilon-P, which was activated in 100% methanol before use. Tris-glycine buffer (see reagents table) was used for transfer, run at 30 V for 90 min on ice. Membranes were blocked with tris-buffered saline with Tween 20 (TBS-T) with 5% bovine serum albumin (BSA) at room temperature for 1 hour and incubated with primary antibodies diluted in TBS-T with 1% BSA overnight at +4°C. Following three TBS-T washes, secondary horseradish peroxidase (HRP) antibody was applied (1:5000 dilution in TBS-T with 1% BSA) for 45 min. Enhanced chemiluminesence was applied after three further TBS-T washes and images were taken using the LICOR Odyssey Imaging System. Protein expression was quantified using ImageJ. Individual bands were quantified, and protein expression was calculated relative to the housekeeper protein using ImageJ. Biological replicates were conducted by obtaining lysates from different passages of each cell line, run on individual gels.

### Histological analysis—Hematoxylin and eosin

Samples were fixed in 4% neutral buffered formalin (NBF) for 12 hours and stored in 70% ethanol before processing and embedding in paraffin wax. To prevent loss of the small tissues during processing each slice was placed into a Cellsafe biopsy insert inside the processing/embedding cassette. Four- to 5-μm microtome sections were generated and mounted on negatively charged slides. Slides were kept overnight at +50°C before rehydration in Citroclear and decreasing concentrations of ethanol. After rehydration, slides were submerged in Meyer’s hematoxylin for 5 min and washed with tap water. Brief washes in acid alcohol, Scott’s tap water, and lithium carbonate followed with distilled water rinse between each step. Dehydration in increasing ethanol (50, 70, and 90%) concentrations preceded 15-s eosin submersion and final submersion in 100% ethanol. To mount samples, slides were first kept in xylene for 1-min intervals before coverslips were mounted with DPX mountant and left to dry overnight.

### Immunohistochemistry

Slides were treated as in “histological analysis” section for IHC up to the point of rehydration in successively decreasing ethanol concentrations and ddH_2_0 submersion. Following rehydration, an antigen retrieval step was performed to improve staining quality. Retrieval was optimized for individual antibodies using a pressure cooker (reaching +125°C for 2 min and +90°C for 1 min before cooling). Staining bins were kept on ice for 1 hour before a hydrophobic PAP pen was used to circle the specimens. Samples were then blocked for 15 min with dual endogenous enzyme block and 45 min with 5% goat serum in TBS to reduce nonspecific binding. Primary antibodies were applied and kept overnight at +4°C in a humid chamber. All antibodies were diluted in antibody diluent. Slides were washed three times before 45-min incubation with secondary antibody HRP. Staining was visualized through short exposure to diaminobenzidine (DAB) diluted 1:50 before the reaction was ceased by submersion in distilled water. Slides were then counterstained with 20-s immersion in hematoxylin Gill, washed, and dehydrated. Following xylene submersion, coverslips were applied using DPX mountant and slides were dried in the fume hood for 48 hours before imaging at 20/40× magnification using the Aperio Brightfield Slide Scanner. Image quantification was carried out using QPath. Tissue was identified using stain vector estimation with deprecated tissue detection used to map tissue area before analysis. Deprecated cell and membrane detection was subsequently used to identify cells with parameters kept constant between samples. Positive cell detection was used to quantify staining intensity within predefined tumor and stroma areas and an average taken across the slice. *H*-score thresholding was kept constant between samples for each individual marker. Three independent patient samples were used for experiments unless stated otherwise.

### RNA extraction and qPCR

RNA extraction and isolation from the avatars were performed using Monarch Total RNA Miniprep Kit. The avatar was initially suspended in 300 μl of DNA/RNA protection reagent from the Monarch Total RNA Miniprep Kit. This was followed by manual dissociation using a pestle and mortar (TaKaRa BioMasher Standard). Manual dissociation was performed for 5 min; the sample was subsequently transferred to a QIAshredder spin column and spun for 2 min at maximum speed. Enzymatic dissociation was performed with proteinase K as per the Monarch Total RNA Miniprep. All subsequent steps were performed exactly as specified by the Monarch Total RNA Miniprep Kit protocol. Residual genomic DNA was removed using on-column deoxyribonuclease I treatment. RNA was converted into cDNA using the LunaScript RT SuperMix kit (by New England Biolabs) as per the manufacturer’s protocol. Luna Universal qPCR Master Mix was used to measure expression of individual genes of interest. Primers were designed online using PrimerBank (*45*). The reactions were performed in duplicate and were run for 45 cycles on a StepOnePlus Real-Time PCR machine. To determine the change in mRNA expression, normalization to β-2-microglobulin was performed followed by the cycle threshold calculation method.

### GeoMX spatial transcriptomic analysis

Spatial transcriptomic analysis was provided by NanoString Technologies Inc. through their GeoMX DSP Technology Access Program Grant. Formalin-fixed paraffin-embedded samples were provided cut at 5 μm thickness and mounted on negatively charged slides. Samples were sent to the NanoString Technology Access Program labs for analysis using the GeoMX Digital Spatial Profiler. Slides were incubated with oligonucleotide-antibody conjugates with photocleavable linkers. Ultraviolet light was then used to selectively release oligonucleotide barcodes, which were read and quantified through sequencing. Four immunofluorescent markers were used for morphology definition, facilitating region of interest (ROI) selection. A total of 68 ROIs were selected across treated and control samples. The Whole Transcriptome Atlas was used for sample profiling, with genes mapped to barcodes using an in-house algorithm produced by NanoString Technologies Inc., generating spatially resolved transcriptional data. Quality control involved assessing raw read threshold, percent-aligned reads, and sequencing saturation. A quantification limit was set based on negative probe signal (mean + two SDs). Reads were filtered based on their expression in >5% ROIs and counts were normalized to account for differences in ROI size and cellularity.

### Multiplexed IF and HALO analysis

The multiplex IF staining of avatars was performed in collaboration with the Oxford Translational Histopathology Lab. Slides were stained using the Leica BOND RXm autostainer machine (Leica, Microsystems) while following the OPAL protocol 28 (AKOYA Biosciences). A total of six staining cycles were subsequently performed. The following primary antibody-opal fluorophore pairing was used: CD4–Opal 520, CD8–Opal 570, CD20–Opal 480, Foxp3–Opal 620, CD68–Opal 690, pan-cytokeratin–Opal 780. In adherence to the manufacturer’s instructions, the primary antibodies were incubated for 1 hour and subsequently detected with the BOND 4 Polymer Refine Detection System (DS9800, Leica Biosystems). The DAB step was replaced by the opal fluorophores, which consisted of a 10-min incubation with no hematoxylin step. The antigen retrieval step was performed with Epitope Retrieval Solution 2 (AR9640, Leica 8 Biosystems) for 20 min at 100°C. This was performed before the application of each primary antibody. VECTASHIELD Vibrance Antifade Mounting Medium with DAPI 10 (H-1800-10, Vector Laboratories) was used to mount each slide. The AKOYA Biosciences Vectra Polaris was used to obtain whole-slide scans and multispectral images (MSIs). A batch analysis of every MSIs was performed using the inForm 2.4.8 software. The final step consisted of fusing the multiple batched analyzed MSIs on the HALO (Indica Labs) software. This resulted in the creation of a spectrally unmixed reconstructed whole image of the avatar. Analysis of the multiplex IF was performed on the Indica Labs HALO (version 3.0.311.407). This software allows for deconvolution of the image by selecting individual fluorophores for analysis. The initial step was teaching the software a Random Forest Classifier module in which the images were segmented into tumor and stromal regions. Manual annotation of the slides was performed to exclude areas with staining artefacts. Cell detection and subsequent phenotyping was performed using the Indica Labs–HighPlex FL version 3.1.0 (fluorescent images). Individual cells were defined by their expression of the specific markers: Tumor (DAPI^+^ panCytokeratin^+^), CD4 helper (DAPI^+^CD4^+^), CD8 cytotoxic (DAPI^+^CD8^+^), T_reg_ (DAPI^+^CD4^+^Foxp3^+^), B cells (DAPI^+^CD20^+^), and Macrophages (DAPI^+^CD68^+^). Proximity and spatial analysis were performed using the relevant software packages within the Indica Labs HALO software using the “proximity analysis” setting.

### Statistical analysis

All statistical analyses were performed using GraphPad Prism version 9.3.1 or Rstudio. All data are represented as means ± SD unless otherwise specified, with significance determined at *P* < 0.05. *P < 0.05, **P < 0.01, ***P < 0.005, and ****P < 0.001. The Shapiro-Wilk and Kolmogorov-Smirnov normalcy tests were carried out and data determined to be normally distributed were analyzed using *T* tests or analysis of variance (ANOVA) for comparing multiple variables. Multiplex IF proximity measurements were analyzed using Cohen’s ds statistic and 95% confidence intervals using the effectsize package in RStudio (*46*).
