# Supplementary material for: Development of human pancreatic cancer avatars as a model for dynamic immune landscape profiling and personalized therapy
Source: Sci Adv. 2024 Jul 5;10(27):eadm9071. doi: 10.1126/sciadv.adm9071 (PMC11225792; doi:10.1126/sciadv.adm9071)
Supplement: Supplementary file 1 — Figs. S1 to S6 Tables S1 to S5 [file sciadv.adm9071_sm.pdf]

Science Advances
AAAS

Supplementary Materials for

Development of human pancreatic cancer avatars as a model for dynamic immune landscape profiling and personalized therapy

Daniel Hughes *et al.*

Corresponding author: Frances Willenbrock, frances.willenbrock@oncology.ox.ac.uk;
Eric O'Neill, eric.oneill@oncology.ox.ac.uk

Sci. Adv. **10**, eadm9071 (2024)
DOI: 10.1126/sciadv.adm9071

This PDF file includes:

Figs. S1 to S6
Tables S1 to S5

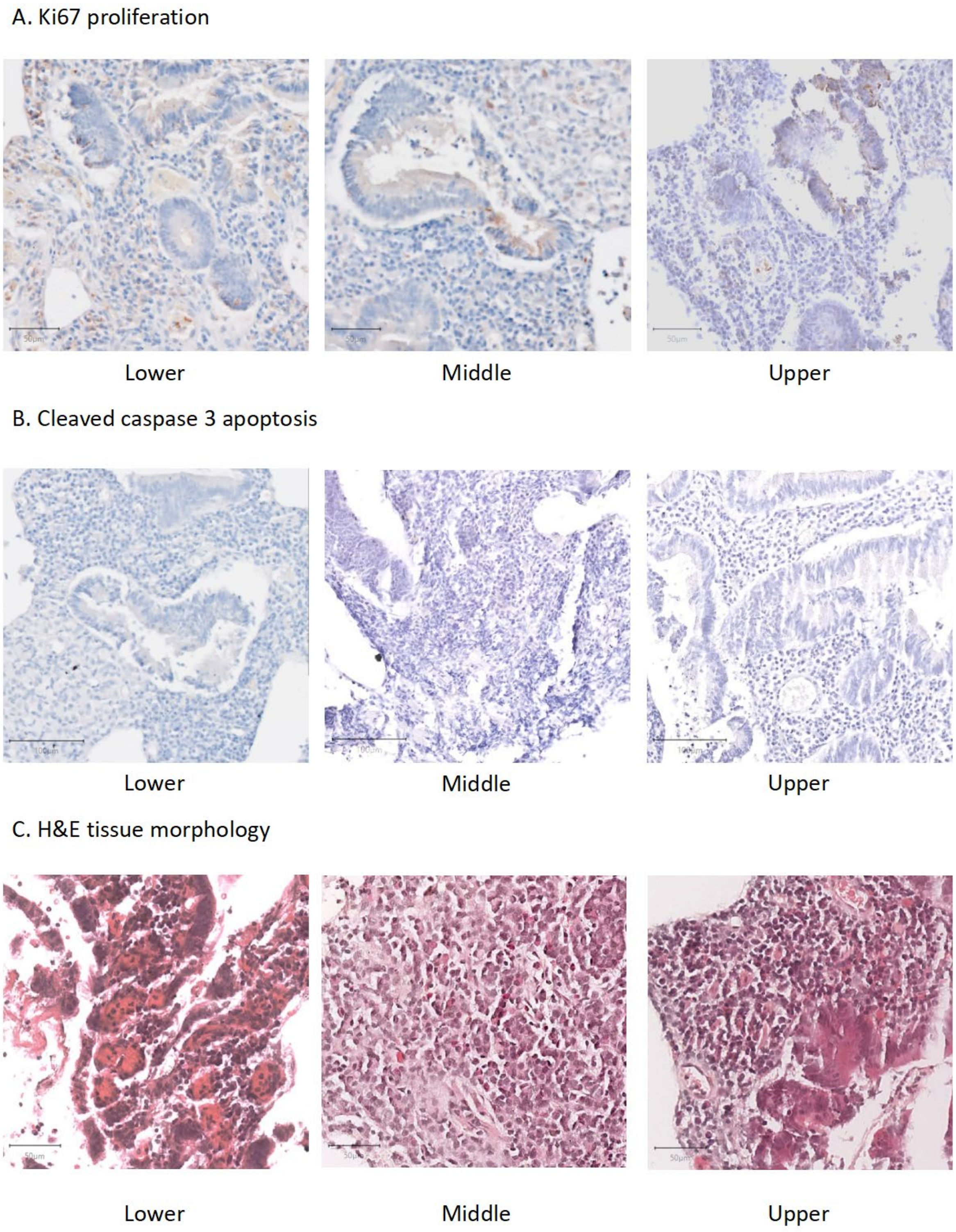

Fig. S1.

Immunohistochemical staining of avatar sections from different tissue locations

Sections are slices taken from the lower, middle and upper sections of a single piece of resection material. Staining is (A) Ki67, (B) cleaved caspase 3 and (C) H & E.

A

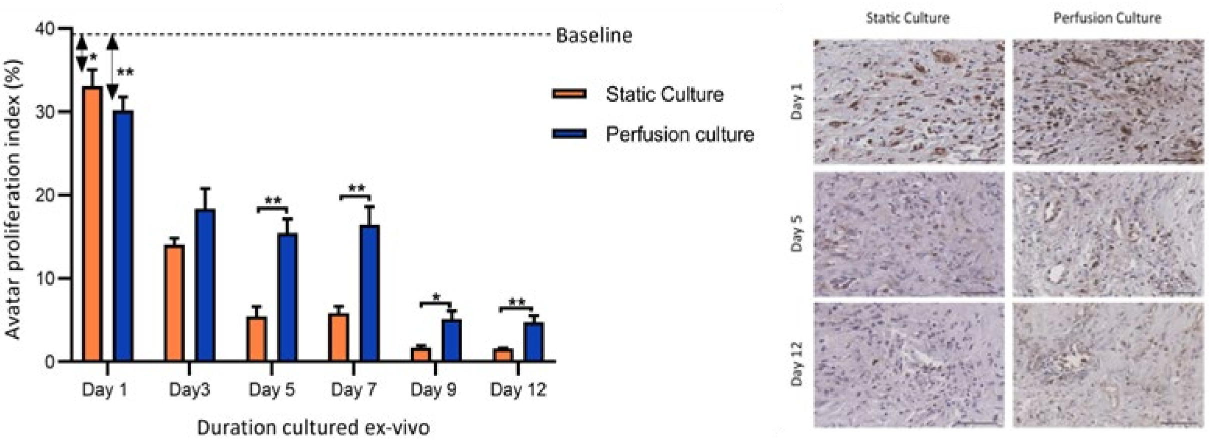

B

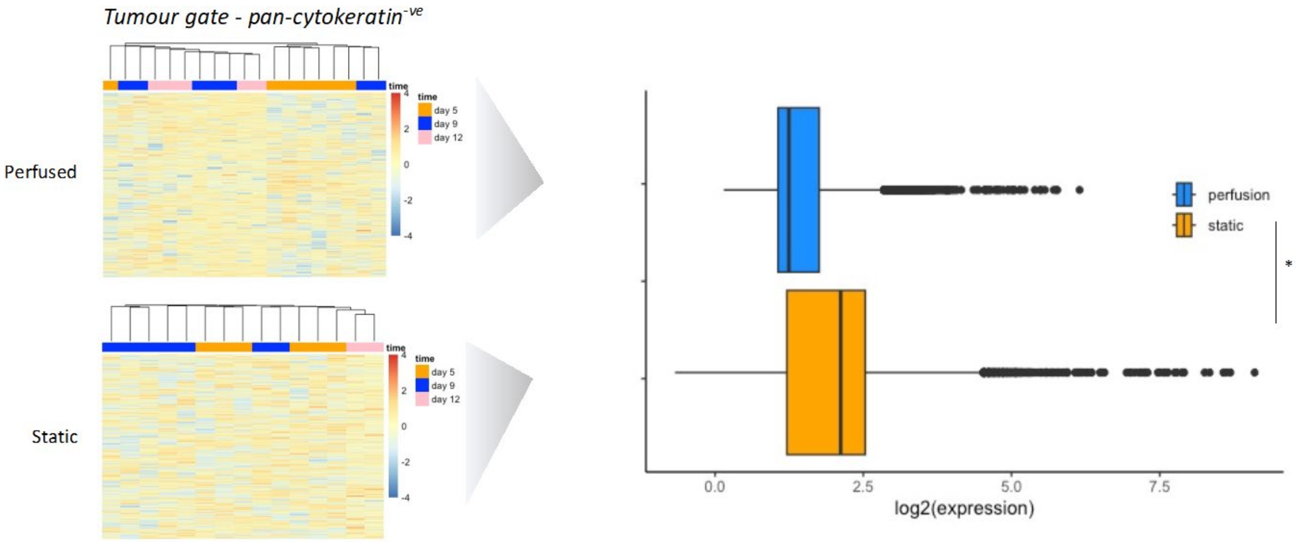

Figure S2

Maintenance of tumour slice integrity over time

(A) Ki67 staining of tumour slices (right as indicated and quantification of 'proliferation index' (left). Value obtained for t = 0 (baseline) is shown by dashed line, (n =3); (B) expression of stromal genes (pan cytokeratin gate) over 12-day time course.

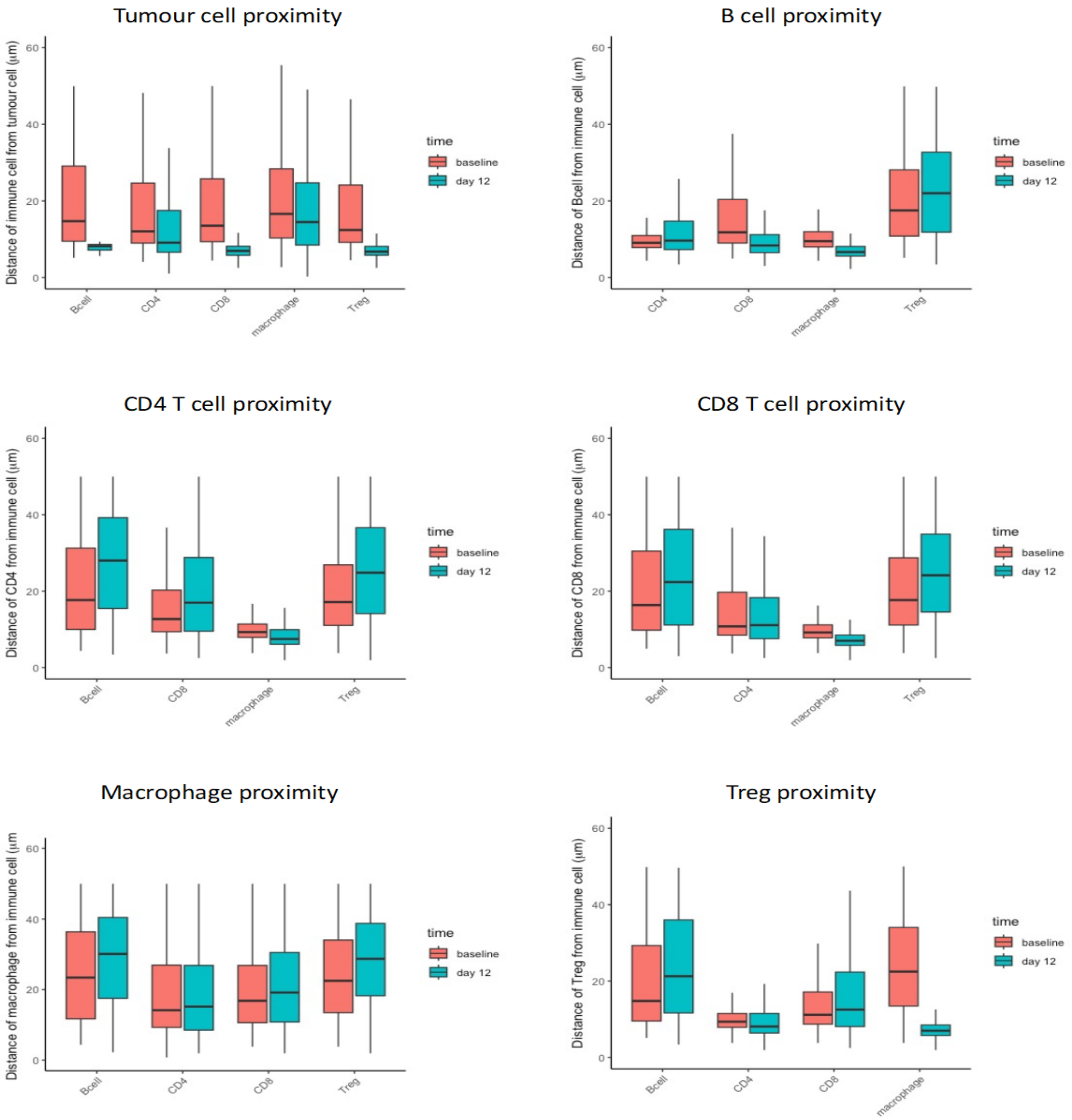

Fig. S3.

Effect of perfusion on cell proximity

Proximity of immune cells to tumour and other immune cells in patient PDCA12. Nearest neighbour analysis was performed on cells that were within 50 mm of tumour cells. Box and whisker plots display the median, quartiles and range of the data. Significance is inferred from a Cohen d statistic for the Welch test of > 0.8

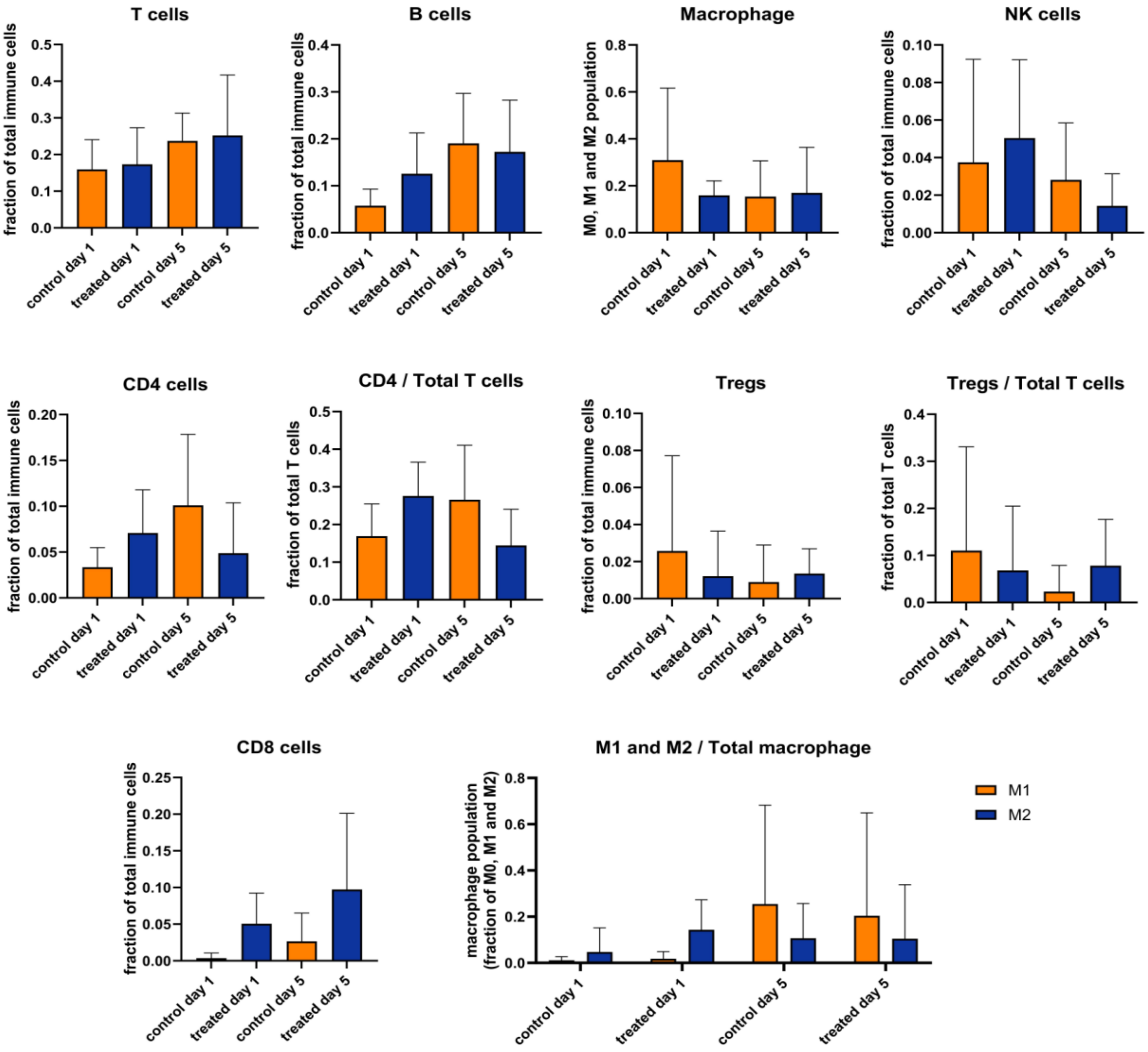

Fig. S4.

Effect of treatment of avatar with metformin and ascorbic acid on immune cell population

The immune component from control (orange) and treated (blue) avatar sections was identified using spatial transcriptomics and deconvoluted using CIBERSORTx.

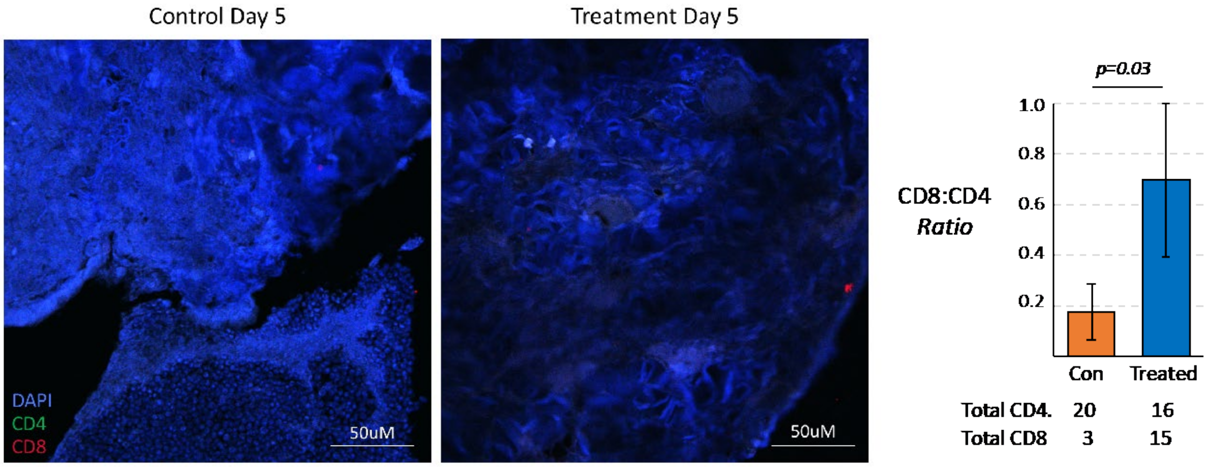

Fig. S5.

Immunofluorescent staining of CD4/CD8 lymphocytes for sections of Metformin/Vitamin C treated tumour slices.

For analysis we took two technical repeats for control and three technical repeats for treated and stained these for CD4 (green) and CD8 (Red). Each image was split into 4 regions and total count of CD4 and CD8 cells collated for orthogonal verification of ratio assessment in Figure 5C. Only images with at least 2 cells were selected (n= 3 control/4 treated) and showed increased CD8:CD4 ratio under treated conditions (p =0.03 one tailed t-test). Total number of cells across image sections used for analysis indicated below.

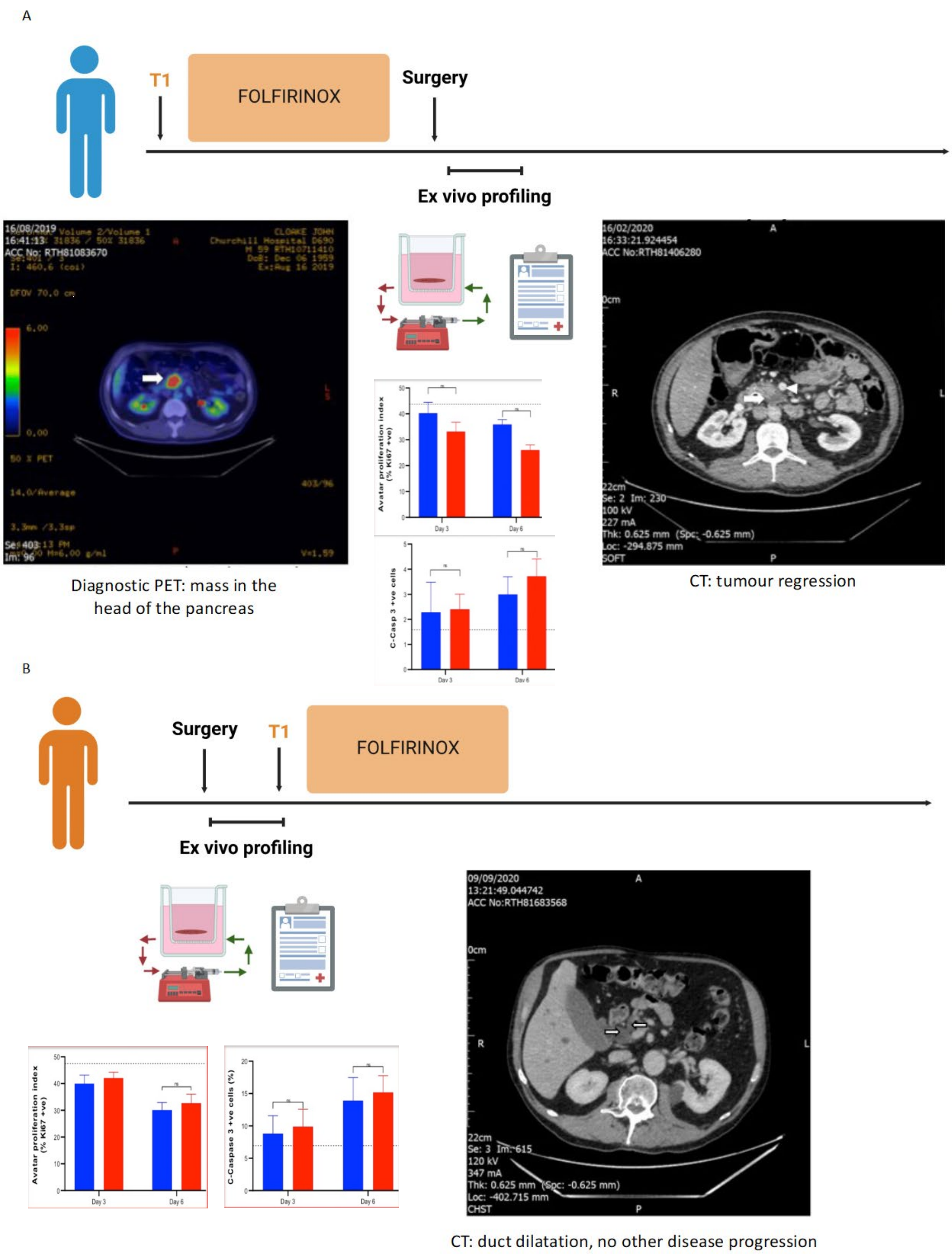

Fig S6

Treatment profile for patients PDCA16 and PDCA17.

Avatar responses to con (blue) or gemcitabine (red) and schematics for the treatment of each patient and accompanying MRI scans.

Target	Supplier	Catalogue number	Concentration
Cleaved caspase 3	Cell Signaling	CST9661	1:200
α-SMA	Sigma Aldrich	A2547	1:30000
Ki67	Millipore	AB9260	1:500
pS6	Cell Signaling	CST2211	1:1000
α-tubulin	Cell Signaling	CST2144	1:1000
Cyclin D1	Abcam	ab134175	1:10000

Target	Forward sequence	Reverse sequence
B2M	CTCCGTGGCCTTAGCTGTG	TTTGGAGTACGCTGGATAGCCT
GATA6	CTCAGTTCCTACGCTTCGCAT	GTCGAGGTCAGTGAACAGCA
KRT81	AATTGAACACCACCTGCGGA	AGTGGGGGATCACACAGAGA

Table S1.
Reagents and primers

A

Cell phenotype	PDCA10		
	Baseline (% Positive cells/all)	D12 static (% Positive cells/all)	D12 perfusion (% Positive cells/all)
CD4 +	4.1	1.5	5.6
CD8 +	3.4	1.9	8.4
Tregs (CD4 + Foxp3 +)	0.2	0.8	0.9
Macrophages (CD68+)	15.8	17.0	18.4
B Cells (CD20 +)	0.9	0.38	1.0

Cell phenotype	PDCA12		
	Baseline (% Positive cells/all)	D12 static (% Positive cells/all)	D12 perfusion (% Positive cells/all)
CD4 +	16.0	9.8	13.1
CD8 +	10.1	3.0	9.1
Tregs (CD4 + Foxp3 +)	3.2	0.2	1.4
Macrophages (CD68+)	44.7	39.5	38.6
B Cells (CD20 +)	2.4	0.1	0.9

Cell phenotype	PDCA13		
	Baseline (% Positive cells/all)	D12 static (% Positive cells/all)	D12 perfusion (% Positive cells/all)
CD4 +	7.6	2.3	9.9
CD8 +	0.7	1.6	5.0
Tregs (CD4 + Foxp3 +)	2.8	0.72	2.0
Macrophages (CD68+)	18.7	11.7	12.7
B Cells (CD20 +)	3.0	0.6	0.6

B

Immune cell- tumour proximity Baseline v day12

	PDCA10		PDCA12		PDCA13	
	Baseline	Day 12 Perfusion	Baseline	Day 12 Perfusion	Baseline	Day 12 Perfusion
Cell phenotype	Average Distance to Tumour cell (um)	Average Distance to Tumour cell (um)	Average Distance to Tumour cell (um)	Average Distance to Tumour cell (um)	Average Distance to Tumour cell (um)	Average Distance to Tumour cell (um)
CD4 +	78.0	75.6	37.9	18.2	32.5	20.1
CD8 +	77.7	50.1	31.8	29.2	97.0	38.0
Tregs (CD4 + Foxp3 +)	253.8	153.4	61.9	57	53.1	63.7
Macrophages (CD68+)	30.3	24.5	13.8	13.6	19.8	27.2
B Cells (CD20 +)	294.5	184.7	251.7	327	60.1	164.2

Table S2.

Results from immunofluorescent staining of avatars using an antibody panel

A, numbers of cells used in the analysis for avatars as a percentage of total immune cells stained in each section; B, proximity of immune cells to tumour cells after 12 days of perfusion culture compared to the baseline numbers

	PDCA10			PDCA12			PDCA13		
Cell type	intra-tumour	peri-tumour	total	Intra-tumour	peri-tumour	total	Intra-tumour	peri-tumour	total
CD4+	344	550	3298	4312	2688	12110	2090	267	3151
CD8+	146	380	2731	2647	1750	7631	54	39	276
Treg	6	21	182	825	534	2458	913	53	1160
Macro phage	1085	1656	12783	9781	7698	33810	1288	1841	7767
B cell	11	46	704	274	278	1846	1103	12	1248

Table S3.
Immune cell numbers.
Intratumour, peritumour (outside but <15um) and total Immune cells and cell areas and close to tumour areas in baseline samples of patients PDCA10, 12 and 13. Graphical representation of this data is in Figure 4C

Number of B cells within 50 µm of tumour or immune cells			
**Target cells**	**PT10**	**PT12**	**PT13**
Tumour cells	571	1367	1240
CD4+	668	1777	1224
CD8+	692	1686	411
Macrophages	702	1836	1223
Tregs	504	1846	1116
**Numbers of CD4+ cells within 50 µm of tumour or immune cells**			
Tumour cells	3020	10873	3085
B cells	982	5705	2288
CD8+	2851	11787	986
Macrophages	3265	12062	3091
Tregs	1259	10814	2501
**Numbers of CD8+ cells within 50 µm of tumour or immune cells**			
Tumour cells	2187	6911	260
B cells	1022	2358	137
CD4+	2123	6989	229
Macrophages	2696	7613	276
Tregs	860	6137	139
**Numbers of Macrophages within 50 µm of tumour or immune cells**			
Tumour cells	10287	31259	7340
B cells	2394	7042	3300
CD4+	8877	26506	5886
CD8+	9686	29932	2330
Tregs	2668	20979	3779
**Numbers of Treg cells within 50 µm of tumour or immune cells**			
Tumour cells	156	2259	1152
B cells	75	895	965
CD4+	159	2410	1129
CD8+	162	2418	339
Macrophages	180	2451	1138

Table S4.

Immune cell proximity to tumour cells and other immune cells

Nearest neighbor analysis was used of cells within 50 m of target cells. Data is depicted in Figure 4

Patient	T stage	N stage	Positive lymph node ratio	M stage	R status	Lymphatic invasion	Vascular invasion	Tumour grade	Stage
PDCA14	pT2	N2	5/18	M0	R0	Yes	No	Moderately differentiated	III
PDCA16	pT2	N0	0/20	M0	R0	No	No	Moderately differentiated	IB
PDCA17	pT1c	N0	0/15	M0	R0	No	No	Moderately differentiated	IB

Table S5

Pathological data from Gemcitabine-treated avatar patients
